# The regional association between bronchiectasis and lung cancer in chest CT

**DOI:** 10.1186/s12890-016-0311-4

**Published:** 2016-11-15

**Authors:** Yeon Wook Kim, Chang-Hoon Lee, Kwang-Nam Jin, Jung-Kyu Lee, Eun Young Heo, Sung Soo Park, Hee Soon Chung, Deog Kyeom Kim

**Affiliations:** 1 Division of Pulmonary and Critical Care Medicine, Department of Internal Medicine, Seoul National University College of Medicine, Seoul, Republic of Korea; 2 Department of Radiology, Seoul National University College of Medicine, Seoul Metropolitan Government-Seoul National University Boramae Medical Center, Seoul, Republic of Korea; 3 Division of Pulmonary and Critical Care Medicine, Department of Internal Medicine, Seoul National University College of Medicine, Seoul Metropolitan Government-Seoul National University Boramae Medical Center, 20 Boramaero-5-Gil, Dongjak-Gu, Seoul 156-707 Republic of Korea

**Keywords:** Bronchiectasis, Lung cancer, Regional association, Lung lobes, Chest CT

## Abstract

**Background:**

Limited studies have examined the association between lung cancer and bronchiectasis (BE). This study evaluated the regional association between BE and lung cancer by analyzing the lobar location of lung cancer in patients with underlying BE.

**Methods:**

This clustered multi-level study enrolled patients who had underlying BE and were newly diagnosed with lung cancer between January 1, 2010 and May 30, 2013 in two referral hospitals in South Korea. By analyzing the presence of lung cancer and underlying BE as event variables at the level of lung lobes on chest computed tomography (CT), we evaluated the association of BE and lung cancer by the locations of the diseases.

**Results:**

Eighty-one patients with BE and combined lung cancer were enrolled. Within 486 lung lobes of the patients, combined BE and lung cancer in the same lobe was found in 11 lobes (2.3 %). Using the general estimating equation assuming BE as a risk factor of lung cancer, the results indicated that the prevalence of lung cancer was significantly lower in the lobes with pre-existing BE (β = −1.09, *p*-value = 0.001).

**Conclusions:**

Regionally, pre-existing BE was associated with a lower risk of the occurrence of lung cancer in the same lobe.

**Electronic supplementary material:**

The online version of this article (doi:10.1186/s12890-016-0311-4) contains supplementary material, which is available to authorized users.

## Background

Chronic inflammation plays a key role in carcinogenesis via disordered necrotic cell death, subsequent epithelial proliferation, and suppressed immunity [1]. Like other organs, chronic inflammation in the lung, such as chronic obstructive pulmonary disease (COPD), is associated with an increased risk of lung cancer, mainly related to repeated airway epithelial injury and accompanied high cell turnover rates [2–4].

Bronchiectasis (BE) is a representative chronic inflammatory airway disease characterized by abnormal and permanent dilatation of the bronchi, accompanied by high levels of inflammatory cytokines [5]. Unlike COPD, however, the impact of the inflammation accompanying BE on lung cancer remains unclear.

Considering reports on the influence of inflammatory signaling in carcinogenesis, chronic inflammation caused by BE might be associated with an increased risk of lung cancer because of the local effects of chronic inflammation caused by repeated airway injury and impaired mucociliary clearance. If this postulate holds, it is reasonable to think that the incidence or prevalence of cancer should be increased close to the pre-existing BE in location [3, 6, 7].

However, there are only limited data on an association between BE and the risk of lung cancer. A few studies reported that patients with BE had elevated levels of serum transforming growth factor-β1 (TGF-β1), which is a potential protective factor against carcinogenesis [5, 8, 9]. In patients with cystic fibrosis showing radiological features of BE, the cystic fibrosis gene mutation was inversely associated with some malignancies [10]. This supports the hypothesis that the association between BE and lung cancer is different from the previously reported role of inflammation in lung cancer carcinogenesis.

The aim of this study was to assess the association between BE and the risk of lung cancer by analyzing the lobar location of lung cancer in patients with underlying BE.

## Methods

### Study design and subjects

**Fig. 1:**
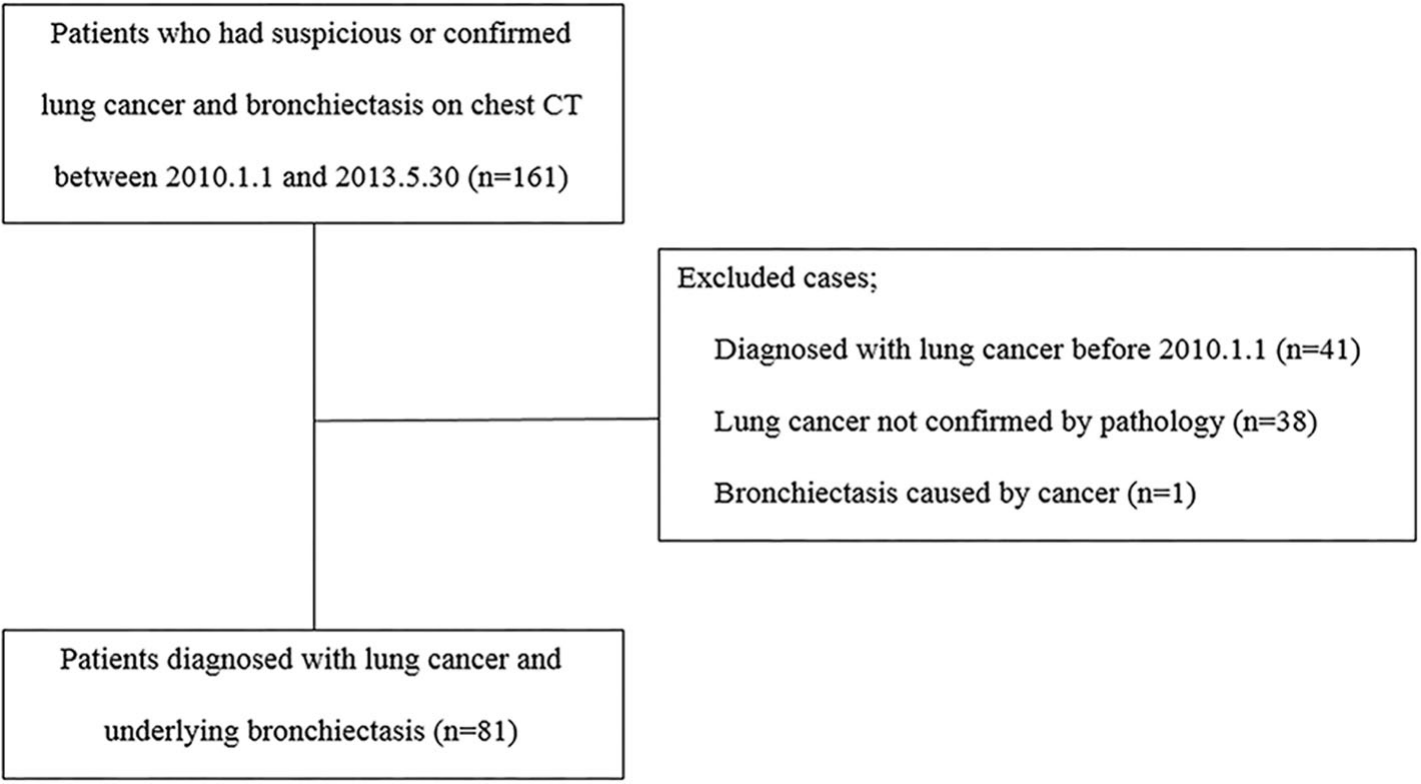
A Flow Diagram of the Study Population

We designed this multi-center-based, retrospective, clustered, multi-level study analyzing the presence of lung cancer and BE as event variables at the level of lung lobes in patients with underlying chronic BE who were newly diagnosed with lung cancer between January 1, 2010 and May 30, 2013. We identified the patients older than 40 years with BE at two referral hospitals in South Korea: Seoul National University Boramae Medical Center and Seoul National University Hospital. Among them, the patients newly diagnosed with lung cancer in the study period were enrolled and analyzed. The flow diagram selecting study population for analyses is shown in Fig. 1. The design of this study was approved by the Institutional Review Board of Seoul National University Hospital (IRB No. H-1312-123-547) and Seoul National University Boramae Hospital (IRB No. 16-2014-137). The institutional review boards waived the need for written informed consent from the participants. Patient records were anonymized and de-identified prior to analysis.

Pre-existing BE was assessed and confirmed with chest computed tomography (CT) analyzed independently by a radiologist and a pulmonologist [11]. If inconsistent findings were found, a consensus decision was reached following a discussion. Patients were excluded if they had BE secondary to mechanical effects caused by lung cancer. Evaluation of secondary BE was based on comparison with previous CTs if available, and inspection of traction and bronchodilation caused by lung cancer (Additional file 1: Figure S1). Reviewing the chest CT findings, the locations of the previous BE and newly developed lung cancer were analyzed among lobes (the lingula of the left lung was considered a distinct lobe, giving six lobes per patient). Demographic data were collected from each patient, including smoking history, baseline spirometric measurements, severity index of BE calculated using the modified Bhalla system and the Reiff score, presence and severity of combined emphysema, tissue type and staging of the diagnosed lung cancer [12, 13].

Among the patients with combined emphysema, the severity of emphysema was assessed visually with CT images according to the modified Goddard scoring system [14, 15]. Six images of three lung slices were evaluated for each patient (the right and left lungs evaluated separately). Each image was classified as normal (score 0), ≤ 5 % affected (score 0.5), ≤25 % affected (score 1), ≤ 50 % affected (score 2), ≤75 % affected (score 3) or >75 % affected (score 4). The average score of six images was considered as a representative value of the severity of emphysema in each patient, and the patients were further categorized into three groups as previously suggested [16]: no/mild emphysema (average severity score < 1); moderate emphysema (1 ≤ average severity score < 2.5); and severe emphysema (average severity score ≥ 2.5).

### Data analysis

Age was given as the median and interquartile range (IQR), while other continuous variables were given as the means with the standard deviations. To evaluate correlations within a subject and within lobes by repeated measures of the events (BE and lung cancer), generalized estimating equations were used and the β-value was calculated. To eliminate any possible interaction that might exist between smoking and BE, analyses were further stratified by the smoking status of the patients. In addition, since emphysema is an important risk factor for lung cancer, subgroup analyses were performed in groups classified according to the presence and severity grade of emphysema. *P*-values < 0.05 were recognized as indicative of statistical significance. All analyses were performed using the SPSS software, version 19.0 (SPSS Inc., Chicago, IL, USA).

## Results

### Baseline characteristics of the study population

Eighty-one patients with pre-existing BE were diagnosed with lung cancer during the study period. The baseline characteristics of the study participants are shown in Table 1. The median age of the study population was 70 (IQR 61–76) years and 52 (64.2 %) were male. Forty-four patients (54.4 %) had a history of exposure to smoking, and 53 (65.4 %) had no or mild emphysema in CT. The baseline spirometric results of the patients at the time of diagnosis with lung cancer included a mean FEV_1_/FVC ratio of 67.7 and mean predicted FEV_1_ of 87.9 %.

**Table 1:** Demographic Characteristics of the Study Population

Characteristics	Patients with bronchiectasis and newly diagnosed lung cancer (*n* = 81)
Age, years, median (IQR)	70 (61–76)
Male, n (%)	52 (64.2)
Smoking history, n (%)	
Current	25 (30.9)
Former	19 (23.5)
Never	37 (45.7)
Smoked pack-year, mean ± SD	21.8 ± 23.7
Presence and severity of emphysema on CT, n (%)	
No/mild emphysema	53 (65.4)
Moderate emphysema	20 (24.7)
Severe emphysema	8 (9.9)
Spirometry, mean ± SD	
FEV1/FVC, ratio	67.7 ± 15.2
FEV1 predicted (%)	87.9 ± 23.4
FVC predicted (%)	91.1 ± 20.9
Charlson comorbidity index, mean ± SD	1.4 ± 1.1

*IQR* interquartile range, *SD* standard deviation, *CT* computed tomography

### Characteristics of diagnosed lung cancer

Seventy-three patients (90.1 %) were diagnosed with non-small-cell lung cancer. Adenocarcinoma was the most common histological type (51.9 %), followed by squamous cell carcinoma (22.2 %) and poorly differentiated carcinoma (7.4 %). When stratified by smoking status, there was significant difference in the histologic type of lung cancer between the current/former smoker group and the never-smoker group (Table 2). The proportion of squamous cell carcinoma and small-cell lung cancer was higher in the current/former smoker group, whereas the proportion of adenocarcinoma was higher in the never-smoker group.

**Table 2:** Histologic Types and Staging of Lung Cancer

Characteristics	Patients with bronchiectasis and newly diagnosed lung cancer (*n* = 81)		*p*-value
	Current/Former smoker (*n* = 44)	Never-smoker (*n* = 37)	
Histologic type, n (%)			0.041
Non-small cell lung cancer	37 (84.1)	36 (97.3)	
Adenocarcinoma	17 (38.6)	25 (67.6)	
Squamous cell carcinoma	14 (31.8)	4 (10.8)	
Poorly differentiated carcinoma	3 (6.8)	3 (8.1)	
Others	3 (6.8)	4 (10.8)	
Small cell lung cancer	7 (15.9)	1 (2.7)	
Staging, n (%)			
Non-small cell lung cancer			0.134
I	12 (27.3)	18 (48.6)	
II	4 (9.1)	2 (5.4)	
III	7 (15.9)	10 (27.0)	
IV	14 (31.8)	6 (16.2)	
Small cell lung cancer			0.285
Limited disease	3 (6.8)	1 (2.7)	
Extensive disease	4 (9.1)	0 (0)	

Thirty patients with non-small cell lung cancer had stage I cancer at the time of diagnosis. Of the eight small-cell lung cancer patients, four were at a limited stage when diagnosed. There was no significant difference in the stage of lung cancer at the time of diagnosis according to the smoking status.

### Regional association between bronchiectasis and lung cancer

To assess the locations of BE and lung cancer, we evaluated 486 lung lobes from 81 patients. Because 4 patients had primary tumors in 2 lobes, the total number of lobes affected with lung cancer was 85. Lung cancer most commonly involved the right upper lobe (5.6 %), followed by the right lower (4.1 %) and left upper lobe (3.9 %) (Table 3). The mean number of lobes affected with BE per patient was 1.7. The mean severity index of BE was 8.0 assessed with the modified Bhalla system and 4.4 assessed with the Reiff score, respectively [12, 13]. BE most commonly involved the left lower lobe (7.4 %), followed by the right upper (5.6 %), right lower (5.6 %), and left upper (4.7 %) lobes. Eleven (2.3 %) lobes contained both BE and lung cancer. There were 123 (25.3 %) lobes with BE only and 74 (15.2 %) with lung cancer only. When using the general estimating equation assuming BE as a risk factor of lung cancer, the calculated β-value was −1.091 (*p* = 0.001) in the analysis of the total lung cancer patients, indicating that the prevalence of lung cancer was significantly lower in lobes with pre-existing BE (Table 4). When analyzing lobes from lung cancer patients separately by smoking status, prevalence of lung cancer remained significantly lower in lobes with pre-existing BE in both the current/former smoker group (β = −1.012, *p* = 0.021) and the never-smoker group (β = −1.152, *p* = 0.011). When evaluating the lobes stratified by the presence and severity of emphysema of the patients, prevalence of lung cancer was significantly lower in lobes with pre-existing BE in patients with no or mild emphysema (β = −1.244, *p* = 0.003). Although not statistically significant, similar trends were shown in the lobes in patients with moderate (β = −0.981, *p* = 0.141) and severe emphysema (β = −0.673, *p* = 0.318).

**Table 3:** Lobar Distribution and Characteristics of Lung Cancer and Bronchiectasis

Characteristics	Total 486 lobes in 81 patients
Affected lobes with lung cancer	
RUL, n (%)	27 (5.6)
RML, n (%)	5 (1.0)
RLL, n (%)	20 (4.1)
LUL, n (%)	19 (3.9)
Lingula, n (%)	1 (0.2)
LLL, n (%)	13 (2.7)
Number of lobes affected with BE per patients, mean ± SD	1.7 ± 0.8
Affected lobes with BE	
RUL, n (%)	27 (5.6)
RML, n (%)	16 (3.3)
RLL, n (%)	26 (5.6)
LUL, n (%)	23 (4.7)
Lingula, n (%)	6 (1.2)
LLL, n (%)	36 (7.4)
BE severity index (modified Bhalla system), mean ± SD	8.0 ± 5.9
BE severity index (Reiff score), mean ± SD	4.4 ± 2.1
Lobes with combined BE and lung cancer, n (%)	11 (2.3)
Lobes with BE only, n (%)	123 (25.3)
Lobes with lung cancer only, n (%)	74 (15.2)

*BE* bronchiectasis, *SD* standard deviation, *RUL* right upper lobe, *RML* right middle lobe, *RLL* right lower lobe, *LUL* left upper lobe, *LLL* left lower lobe

**Table 4:** Risk of Lung Cancer in Patients with Underlying Bronchiectasis Estimated by General Estimating Equation^a^

Population	β-value	95 % CI	*p*-value
Total	−1.091	−1.716 - -0.466	0.001
Classified by smoking history			
Current/Former smoker	−1.012	−1.869 - -0.155	0.021
Never-smoker	−1.152	−2.043 - -0.261	0.011
Classified by emphysema severity			
No/mild emphysema	−1.244	−2.075 - -0.412	0.003
Moderate emphysema	−0.981	−2.287 - 0.325	0.141
Severe emphysema	−0.673	−1.995 - 0.649	0.318

^a^ β-value calculated by evaluating bronchiectasis as a risk factor of lung cancer

## Discussion

This study assessed the association between pre-existing BE and newly diagnosed lung cancer in terms of the lobar distribution. It revealed that the presence of pre-existing BE was associated with a significantly lower risk of lung cancer in the same lobe. These results are interesting and suggest another aspect of the relationship between chronic inflammatory airway diseases and lung cancer. In addition, our data also provide information about the prevalent location of lung cancer and BE in patients which the two diseases coexist. Compared to lung cancer, which most commonly involved the right upper lobe, BE most commonly involved the left lower lobe. To our knowledge, this would be the first description of the association between BE and lung cancer by location of the disease detected using CT at the level of lung lobes [6, 17].

There is limited evidence for the pathophysiological mechanism of the protective effect of BE in local carcinogenesis shown in our study. However, indirect biological plausibility exists. There are reports of elevated serum TGF-β1 levels in patients with BE [5]. In normal and premalignant cells, TGF-β enforces homeostasis and tumor-suppressive effects by regulating cell-autonomous cytostasis, differentiation, and apoptosis. In addition to its direct inhibitory effects, TGF-β can restrict epithelial cell proliferation and carcinogenesis by blocking the production of paracrine factors in the stromal cells [8, 9]. Other plausibility includes the *CTFR* gene. A case–control study suggested that the ΔF508 deletion in the *CFTR* gene in patients with cystic fibrosis, which shows radiological features of BE, is an important protective variant for lung cancer risk [10]. However, more research is needed to identify a biological mechanism that can clearly explain our findings.

Since previous studies report a positive association between the chronic inflammation in COPD or smoking and the risk of squamous cell carcinoma, it is notable that the majority of patients in our study were diagnosed with adenocarcinoma [2, 18]. Moreover, among the patients diagnosed with lung cancer, 46 % were never-smokers, and 65 % had no or mild emphysema. The high proportion of adenocarcinoma, never-smokers, and non-emphysematous subjects among the diagnosed lung cancer patients suggests that the majority of lung cancers in patients with pre-existing BE occurs independently of chronic inflammation. These findings can support our theory that the chronic inflammation caused by BE is not associated with an increased risk of lung cancer.

Previous epidemiological reports have aimed to evaluate the association between prior lung diseases and the risk of lung cancer. They showed that some chronic respiratory diseases, including bronchitis and emphysema, are positively associated with the risk of lung cancer [17, 19]. However, these efforts focused mainly on evaluating diseases and chronic inflammation associated with smoking, a major contributor to lung cancer. To date, the mechanism of airway inflammation in BE, and the association between BE and lung cancer remains unclear. Recently, a nationwide cohort study from Taiwan reported that patients with underlying BE had a 2.36-fold increased risk of lung cancer compared to patients without BE. However, this study has limitations in confirming the disease status only by the diagnosis codes provided from the registered hospitals, and including only inpatients as subjects diagnosed with BE, who had significantly higher rate of comorbidities possibly related to cancer. Moreover, the study used a database that did not contain information on smoking status of the participants, which is a possible strong confounding factor when evaluating risk of lung cancer. The study also could not provide any information about the severity or location of BE and the histological type of lung cancer, which is important when discussing the possible effects of BE and inflammation on the risk of lung cancer [20]. Although our study included a relatively small number of subjects, we were able to obtain detailed information about the diagnosed BE and lung cancer for each patient, and the data on the location of BE and lung cancer made it possible to perform analyses within levels of lung lobes. Results of our study are in concordance with a recent case–control study from South Korea which reported that the concomitant presence of BE was associated with a lower risk of lung cancer in COPD patients [21]. Although BE is a representative chronic inflammatory airway disease causing permanent dilatation of the bronchi and is accompanied by high levels of inflammatory cytokines, our results suggest that the chronic inflammation caused by BE might produce different cytokines compared with other airway diseases and acts in a different way in carcinogenesis of the lung.

The main strength of our study is that the presence of lung cancer and BE was assessed with chest CT, a precise means of evaluating the location of the disease at the level of the lung lobes. In addition, because we analyzed the association between the two diseases by location within patients known to have both diseases, the possible confounding factors of the risk of lung cancer in each patient (*e.g*., age, gender, and smoking status) could be ignored in our study.

To interpret our results correctly, we should consider the limitations of this study. First, given its retrospective design, by selecting patients who were diagnosed with lung cancer, there might have been selection bias enrolling patients at higher risk of, or more susceptible to, lung cancer. However, since the study design was powered to evaluate the association between BE and lung cancer by the locations of the two diseases within each subject, rather than an epidemiology study evaluating a certain population, possible selection bias is not expected to have contributed to the significant results of our study. Second, our data contains no detailed clinical information on the history of BE (e.g. frequency of exacerbations, type of infection, length of follow up, and received treatments), which can be an important issue when discussing the role of chronic inflammation in BE. Lastly, due to the limited data collected on the field of inflammation and carcinogenesis related BE, the validity of the potential explanations on our findings and the role of chronic inflammation might be low. Nevertheless, considering the limited data and difficulty to perform large-scaled studies on the association between BE and lung cancer, our results still would provide novel and relevant information, and motivation for further studies.

Although this study was not a longitudinal study following BE patients, it is reasonable to believe that in the patients evaluated in our study, BE preceded the development of lung cancer since BE is generally accepted to be a chronic condition following infection in childhood, while lung cancer usually develops within years of the initial diagnosis [22]. Moreover, patients were excluded if they had BE secondary to mechanical effects caused by lung cancer. Therefore, despite the retrospective design, we insist that our results represent a temporal relationship between BE and lung cancer, rather than a coincidental finding.

## Conclusions

Regionally, pre-existing BE was associated with a lower risk of the occurrence of lung cancer in the same lobe.

## Additional file

Additional file 1:
**Figure S1.** Example CT images of (A) underlying bronchiectasis and newly diagnosed lung cancer existing in the same lobe and (B) secondary traction bronchiectasis caused by lung cancer. (ZIP 7247 kb)

## Abbreviations

BE: Bronchiectasis
COPD: Chronic obstructive pulmonary disease
CT: Computed tomography
IQR: Interquartile range
LLL: Left lower lobe
LUL: Left upper lobe
RLL: Right lower lobe
RML: Right middle lobe
RUL: Right upper lobe
SD: Standard deviation
TGF-β1: Transforming growth factor-β1
